# Advanced glucose control strategies leveraging Raman spectroscopy for optimized mammalian cell culture manufacturing

**DOI:** 10.1002/btpr.88531

**Published:** 2026-06-29

**Authors:** Matthew Banner, Daniel Ray, Irfan Ahanger, Fabio Zurlo, Noah Hitchcock, Rajesh Mistry, Kasia Kozakowska‐McDonnell, Matthew Cheeks, Richard Turner, Jonathan Welsh, Suzanne S. Farid, Michael Thomas, Stephen Goldrick

**Affiliations:** ^1^ The Advanced Centre of Biochemical Engineering, Department of Biochemical Engineering University College London London UK; ^2^ Analytical Sciences, Biopharmaceutical Development, R&D AstraZeneca Cambridge UK; ^3^ Cell Culture and Fermentation Sciences, Biopharmaceutical Development, R&D AstraZeneca Cambridge UK; ^4^ London Centre for Nanotechnology University College London London UK

**Keywords:** biotherapeutics, machine learning, mammalian cell culture, process analytical technology, process control, Raman spectroscopy

## Abstract

Maintaining consistent quality in the manufacturing of biotherapeutic proteins in mammalian cell culture is challenging, with unplanned deviations causing inconsistencies and potential batch failure. Current methods for monitoring and controlling critical process parameters (CPPs) rely on slow, labor‐intensive offline analyses. This is particularly problematic in upstream manufacturing, where infrequent measurements hinder real‐time CPP monitoring and result in suboptimal control. This study developed and implemented a robust, standardized framework that integrates Raman spectroscopy with real‐time machine learning models and bioreactor control via OPC‐UA, enabling seamless communication and effective control of mammalian cell culture CPPs. High‐accuracy ML models (*R* ^2^ >0.92) enabled real‐time monitoring of glucose, lactate, viable cell density, and antibody titre. The glucose model was integrated into an automated process analytical technology (PAT) control strategy to maintain glucose at a predefined setpoint. The PAT strategy was compared to a manual bolus glucose control under high and low glucose feeding regimes. The PAT control approach observed an increase in biotherapeutic product titre by up to 35% and reduced glycation by up to 27%. These results indicated that both glucose setpoints and fluctuations impacted cell culture performance. In conclusion, this study highlighted the value of PAT tools in automating control loops, consequently improving process performance with enhanced productivity and quality.

## 1 INTRODUCTION

Protein‐based biotherapeutics, used for treating a wide range of diseases and diagnostics, are complex and challenging molecules to manufacture in mammalian cell culture processes. A significant challenge in manufacturing these molecules is ensuring consistency in the expression, structure, and therapeutic efficacy properties. This variability arises from multiple factors, including the protein primary structure, such as the amino acid sequence,^1^ as well as the choice of host cell line^2^ and clone selection.^3^ Additionally, manufacturing process conditions across unit operations have been documented in the literature to contribute toward this variability.^4^, ^5^, ^6^, ^7^ Variability in biotherapeutic properties can impact the pharmacokinetics, safety, and efficacy of the molecule.^8^ Consequently, there is significant industry interest in reducing this variability within appropriate ranges to ensure consistent product performance. A subset of these properties, known as critical quality attributes (CQAs), define the safety, efficacy, and overall consistency of the biotherapeutic.

In upstream manufacturing, many CQAs are influenced by the manufacturing conditions within the bioreactor including glycosylation, aggregation, fragmentation, charge variants, and glycation.^9^, ^10^ An example is the impact of large fluctuations in glucose concentration caused by the feeding regime deployed in fed‐batch cultures. Variations in the glucose concentration have been shown to affect product titre and glycosylation.^11^

The process conditions which directly impact CQAs are referred to as critical process parameters (CPPs). Examples of these include temperature and pH. One method to improve consistency in biotherapeutic quality is to increase the frequency of measurements for monitoring and employ tighter control strategies on these CPPs to reduce their impact on their respective CQAs. The majority of process parameters including CPPs are usually monitored using offline analytical techniques, such as metabolite analyzers for measuring pH, glucose, and lactate concentrations. These techniques often require significant time and labor to consistently monitor throughout the manufacturing process. The ability to monitor and control these process parameters in a more immediate manner remains a challenge in the sector.

There was a directive from regulatory bodies to adopt and incorporate process analytical technology (PAT) to improve process monitoring and control throughout the manufacturing process.^12^ These technologies provided inline supplementary information regarding the unit operation but require extensive data analysis and preprocessing to convert the data generated from these sensors into useful process insights. One particular PAT sensor, garnering interest in the biomanufacturing sector, is Raman spectroscopy. This technology can capture structural information on a wide range of molecules and is resistant to interference from water in comparison to other spectroscopy techniques such as near infrared (NIR) and infrared (IR).^13^ Raman spectroscopy in conjunction with machine learning (ML) techniques has enabled the real‐time monitoring of various upstream process parameters.^14^, ^15^, ^16^ Rafferty et al. used partial least squares regression (PLSR) and Raman spectroscopy to predict a broad range of offline cell‐related parameters including viable cell density (VCD), cell viability, cell diameter, and viable cell volume.^17^ They also developed a model to predict capacitance measurements, highlighting the versatility of Raman spectroscopy in predicting a diverse set of parameters and as an alternative for other PAT tools. The process insights gained from Raman spectroscopy are not limited to process parameters. A study by Liu et al. used Raman spectroscopy and PLSR for monitoring a range of CQAs from an inline perfusion cell culture process.^18^ These PLSR models were able to predict structural information about the therapeutic product such as glycosylation in the form of percentage of mannose 5 and both aggregation and fragmentation information including the percentage of high and low molecular weight species. Furthermore, these tools have been adopted into control strategies in recent years, with multiple applications reported.^19^, ^20^, ^21^, ^22^, ^23^, ^24^, ^25^, ^26^

However, widespread implementation remains limited due to challenges in the diversity of equipment, software limitations and regulatory compliance such as 21 CFR Part 11. As the number of available PAT and manufacturing control systems increases, so too does the number of proprietary software solutions, each employing unique methods for data storage and transfer. While this expansion offers greater flexibility, it also creates interoperability challenges, requiring significant resources to ensure communication across devices. A major barrier to interoperability is the variety of operating systems (OS) and legacy platforms used in manufacturing, which can lead to compatibility and security issues in data exchange. To address this, many systems are now employing cross‐platform machine‐to‐machine communication protocols, such as Open Platform Communications Unified Architecture (OPC‐UA), to enable secure and standardized data exchange between different systems.

Despite these advancements, few commercial software solutions currently support communication between PAT and manufacturing equipment, whether via OPC‐UA or alternative methods. While these solutions offer limited modeling flexibility and constrain the volume of data that can be exchanged, they remain accessible and good manufacturing practice (GMP) compliant. GMP regulations, 21 CFR Part 11, require manufacturers to maintain electronic records and signatures that ensure data authenticity, reliability, and integrity. However, for data‐intensive PAT systems, the vast amount of generated data presents a significant challenge in infrastructure and storage capacity, making compliance a key concern for manufacturers.

This paper proposes a novel solution by introducing a robust and standardized framework for implementing PAT devices and bespoke predictive models to control a mammalian cell culture system. The innovative framework facilitates the communication and exchange of information between 3 systems through an OPC‐UA framework: a PAT sensor (Raman Spectrometer), a Raspberry Pi and bioreactor control system. The methodology for developing predictive ML models for real‐time monitoring of multiple process parameters from inline Raman spectroscopy data is outlined. This is followed by the daily workflow for a bolus‐shot glucose control strategy and the PAT feedback control strategy using this robust framework. Finally, a comparative analysis of these feedback control strategies was conducted using different glucose feeding strategies to evaluate their impact on culture performance and product characteristics.

## 2 MATERIAL AND METHODS

### 2.1 Cell line and subcultures

The cell line used was Chinese Hamster Ovary (CHO) producing a biotherapeutic protein. The cell line was cultured in chemically defined CHO media at 36.5°C, 70% humidity and 6% carbon dioxide, shaken at constant rpm, and passaged 2–3 times a week for propagation and bulked up for inoculation into the bench‐scale bioreactors. All cell lines and molecules used in this study were developed and supplied by AstraZeneca, Cambridge, UK.

### 2.2 Mammalian cell culture process

Seven mammalian cell cultures were grown in 7 L glass bioreactors (Applikon, the Netherlands) with a working volume of 5 L at a seeding density of 1E6 cells/mL. The cell cultures were controlled using a DASware controller (Eppendorf, Germany). The dissolved oxygen (DO) set point was controlled at 50% saturation using a cascade gassing strategy and impeller agitation speed. The temperature and pH of all bioreactors were controlled at 35.5°C and pH 7.0 with a deadband of ± 0.1 respectively. The pH was controlled through a combination of sparging of CO_2_ gas and the addition of sodium carbonate base to maintain within the deadband. 5 mL of antifoam was added to the bioreactor every day to reduce excess foaming. All bioreactors received identically scheduled two‐part nutrient‐dense feeds on a fixed 2‐day cycle to promote cell growth and increase protein expression. These feeds contain a wide range of amino acids. The bioreactors were covered in aluminum foil to minimize the impact of ambient light on Raman spectral acquisition. For each bioreactor, a 15 mL sample was taken before and after the glucose bolus addition. For both samples, glucose concentration, lactate concentration, and ammonia concentration were measured using a Nova FLEX analyzer (Nova Biomedical, USA) in addition to VCD and cell viability using the Vi‐Cell automated cell viability analyzer (Beckman Coulter, USA).

Bioreactors 1–5 were cultured using an offline glucose control strategy (Manual control). In this approach, offline glucose samples were used to trigger the addition of glucose bolus shots up to the setpoint if the measurement was below the trigger. Bioreactors 1, 2, 3, and 4 followed a higher glucose setpoint, referred to in this study as the high glucose feeding strategy (High). In this strategy, the glucose setpoint was defined as 6 g/L from day 2 to day 5 and 8 g/L from day 6 to day 14, with the trigger point being 4 and 6 g/L, respectively. Bioreactor 5 used a lower glucose setpoint, known as the low glucose feeding strategy (Low). Here, the glucose setpoint was defined as 4 g/L from day 2 to day 5 and 6 g/L from day 6 to day 14, with the trigger point being 2 and 4 g/L, respectively.

Two bioreactors were operated utilizing the PAT feedback control strategy with an adapted version of both the Low and High glucose feeding strategies. As a result of the increased measurement frequency, the glucose setpoints for both strategies were adjusted so the trigger point and the glucose setpoint were the same. For the Low glucose strategy, the setpoint was 2 g/L from day 2 to day 5 and 4 g/L from day 6 to day 14. For the High glucose strategy, the setpoint was 4 g/L from day 2 to day 5 and 6 g/L from day 6 to day 14.

### 2.3 Titre analysis

From day 3 onwards, daily cell culture supernatant samples were collected for quantification of product titre using a protein A high‐performance liquid chromatography on an Agilent 1200 (Agilent Technologies, USA) by comparing the sizes of the peaks from each sample with a calibration curve, where protein A binds to the CH_3_ region of the Fc domain of the therapeutic protein, as described in Sou et al.^27^

### 2.4 Glycation analysis

Culture supernatant samples were taken for glycation analysis on day 6, 8, 10, 12, and 14 before and after glucose addition. The samples were deglycosylated using peptide‐N‐glycosidase F (PNGase F) (Promega) and analyzed on a reverse phase Waters ACQUITY ultra‐performance liquid chromatography (UPLC) Protein BEH C4 column connected to a Waters Acquity UPLC coupled to an ESI‐Orbitrap MS instrument (ThermoFisher, USA) at 65°C. Mobile Phase A (0.01% TFA and 0.1% FA in Water) and Mobile Phase B (0.01% TFA and 0.1% FA in Acetonitrile) were used, and the sample was separated and eluted from the column by applying a gradient of increasing mobile phase B. Intact mass parameters were set to acquire MS data. The measured mass of the molecule and additional post‐translational modifications are obtained through the deconvolution of mass spectra using the intact mass module from Byos PMI (Protein Metrics Inc., USA) and compared to the theoretical mass of the molecule based on the amino acid composition. Glycation levels were quantified by comparing intensity units of unmodified protein versus glycated species.

### 2.5 Raman spectral acquisition

All spectral acquisitions were captured using a Kaiser RNX2 Raman spectrometer (Endress & Hausser, USA) with the bIO‐LAB immersion probe. The system was equipped with a 785 nm laser with a spectral range of 100–3425 cm^−1^, recording spectra with an exposure time of 15 s and 50 accumulations, resulting in a total collection period of 12.5 min per vessel.

### 2.6 Spectral preprocessing and model building

The Raman data collected by the RNX2 Raman spectrometer was preprocessed using Python 3.8. For all models, the spectral region between 400 and 2000 cm^−1^ was used for all preprocessing. The fluorescent background was approximated and removed from the raw spectra using a choice of baseline correction techniques. The subsequent baseline corrected spectra were normalized through utilizing a standard normal variate algorithm to improve spectra comparability between runs. Further, the normalized spectra were smoothed using a Savitzky–Golay smoothing filter to reduce the impact of random noise on the spectra. Upon preprocessing, PLSR was used to develop models from the spectra to predict glucose concentration, lactate concentration, VCD, and antibody titre. Bioreactors 1, 3, 4, 5 were used to train and cross‐validate each PLSR model, while Bioreactor 2 was set aside as a testing set to assess the model's ability to perform on unseen data. The choice of preprocessing methods and hyperparameters was identified through minimizing the average root mean squared error (RMSE) of a four‐fold cross‐validation method. The optimal preprocessing techniques and hyperparameters for each process parameter model are depicted in Table 1.

**TABLE 1:** Summary of the chosen preprocessing techniques, preprocessing hyperparameters and model hyperparameters for the partial least squares regression (PLSR) models produced for glucose concentration, lactate concentration, viable cell density (VCD) and antibody titre.

Response variable	Baseline correction method	Scatter correction method	Savitzky–Golay filter parameters	PLSR number of latent variables
Glucose concentration (g/L)	Polynomial baseline correction Polynomial Order: 6	Standard normal variate	Window length: 49 Polynomial order: 3	5
Lactate concentration (g/L)	Polynomial baseline correction Polynomial order: 4	Standard normal variate	Window length: 45 Polynomial order: 2	5
Viable cell density (×1E6 Cells/mL)	Asymmetric least squares Smoothness parameter: 100 Asymmetry parameter: 0.01	Standard normal variate	Window length: 29 Polynomial order: 4	8
Antibody titre (mg/L)	Asymmetric least squares Smoothness parameter: 1000 Asymmetry parameter: 0.01	Standard normal variate	Window length: 29 Polynomial order: 3	3

### 2.7 Partial least squares regression model

To predict the cell culture process parameters, PLSR was used. This approach looks for patterns in complex data (in this case, spectra measurements) and relates it to specific outcomes, for example, glucose levels or viable cell concentration. PLSR is a commonly used technique to model Raman spectra and other high‐dimensionality applications.

The method works by breaking down both the measurements and the outcomes into a smaller number of building blocks (latent variables) to make the relationship between them easier to find. Once the PLSR model finds this relationship, a formula is generated which can be used to quickly and accurately predict what is happening in the cell culture from new measurement data.

The PLSR model was generated using the nonlinear iterative partial least squares algorithm described in detail by Wold et al.^28^ Initially the preprocessed spectra (X) were decomposed into N latent variables. Similarly, the process parameter (Y) was decomposed into N latent variables (Equations (1) and (2)).

Decomposition of *X* into *N* latent variables.

(1) $$X=TP^{\prime }+\varepsilon_{X}$$

where T is the matrix of scores, P is the matrix of loading and $\varepsilon_{X}$ is the matrix of errors generated by decomposing X into N latent variables.

Decomposition of *Y* into *N* latent variables.

(2) $$Y=UQ^{\prime }+\varepsilon_{Y}$$

where U is the matrix of scores, Q is the matrix of loading and $\varepsilon_{Y}$ is the matrix of errors generated by decomposing Y into N latent variables.

B is defined as the inner relationship matrix which is generated by relating X to Y through the Equation (3):

Formula for inner relationship matrix for PLSR application.

(3) $$B=X^{\prime }T{\left(X^{\prime }X\right)}^{-1}$$

The PLSR model is iterated for each latent variable, until convergence is achieved. Once achieved, a matrix of regression coefficients, β, is generated using Equation (4):

Formula of the regression coefficient matrix of PLSR.

(4) $$\beta =W{\left(P^{\prime }W\right)}^{-1}\mathrm{diag}\left(B\right)$$

With the weight matrix, W, calculated iteratively to maximize the covariance between X and Y by projecting X onto N latent variables.

The cumulative sum of these regression coefficients predicts the response variable(s) of interest, $\hat{Y}$, calculated using Equation (5) as:

PLSR response variable formula.

(5) $$\hat{Y}=\mathrm{X\beta}$$

where β, accounts for contributions from all the latent variables, N.

### 2.8 PLSR Prediction interval

No model is perfect. When a model is used to predict a value, there is always some uncertainty. Prediction intervals quantify a range within which a given future observation is expected to fall based upon the model and associated uncertainty.

Calculating and estimating the prediction interval can be complex for PLSR models due to the projection of the original predictor variables onto a small number of latent variables, which can alter the error structure and invalidate assumptions used in ordinary least squares regression.^29^

To date, there are multiple methods to approximate a prediction interval for PLSR, including: Bootstrapping, Jack‐knifing, and Local Linearization.^30^, ^31^, ^32^

In this paper, the method originally developed by Nomikos et al. was applied to approximate a prediction interval around the PLSR model predictions.^29^ This method was chosen because of its computational simplicity and ease of deployment compared to other techniques.

When a new observation is analyzed, a corresponding model prediction, ${\hat{y}}_{\mathrm{new}}$, is calculated based on the new preprocessed spectra, $X_{\mathrm{new}},$ and PLSR coefficients, β using Equation (6).

New PLSR prediction formula.

(6) $${\hat{y}}_{\mathrm{new}}=X_{\mathrm{new}}\beta$$

However, the new prediction is an estimation that does not consider the uncertainty of the model in the calculation. The mean squared error of the training (MSE) set used to develop the model has been shown to estimate the uncertainty associated from the model and sample terms.^33^ This can be estimated using Equation (7).

Training mean squared error formula for PLSR.

(7) $$\mathrm{MSE}=\frac{\sum_{i=1}^{n}{\left(y_{i}-{\hat{y}}_{i}\right)}^{2}}{n-N-1}$$

where ${\hat{y}}_{i}$ is the predicted response at observation i, $y_{i}$ is the corresponding measured offline concentration, both from the training set, n is the total number of observations in the training set, N is the number of latent variables in the PLSR model.^33^

To assess and quantify the influence on a new observation of the model predictions, a metric of the distance of the new observations from the training set is selected. This metric, referred to as the leverage term ($h_{\mathrm{new}}$), weighs observations which are further away from the center of training set greater. The leverage term is defined in Equation (8).

Leverage for new observation formula.

(8) $$h_{\mathrm{new}}=t_{\mathrm{new}}{\left(T^{\prime }T\right)}^{-1}t_{\mathrm{new}}^{\prime }$$

where $\;t_{\mathrm{new}}$ is the score for the new observation project into latent space, $t_{\mathrm{new}}=X_{\mathrm{new}}W{\left(P^{\prime }W\right)}^{-1}.$ ^34^

With all the relevant terms, an estimation for the prediction interval around a new model prediction can be computed as follows:using Equation (9).

Prediction interval construction.

(9) $$\mathrm{PI}={\hat{y}}_{\mathrm{new}}\pm t_{\;n-N-1,\;\frac{\alpha }{2}}\times \;\sqrt{\mathrm{MSE}}\;\sqrt{\left(1+h_{\mathrm{new}}\right)\;}$$

where $\;t_{n-N-1,\;\frac{1-\alpha }{2}}$ is the critical value of the Studentized *t*‐distribution with degrees of freedom defined as n−N−1 at 1−α confidence level.^29^ This study utilized a 99% (α=0.01) prediction interval.

## 3 RESULTS AND DISCUSSION

This paper evaluated inline Raman spectroscopy as a tool to monitor and control a mammalian cell culture process for the expression of a biotherapeutic product. Initially, Raman spectroscopy was assessed as a tool to predict four different cell culture parameters inline through the use of extensive preprocessing and ML techniques. These parameters included glucose concentration, lactate concentration, VCD, and antibody titre. Subsequently, the glucose prediction model was deployed into a PAT control strategy to maintain the glucose concentration at a predefined setpoint in a mammalian cell culture. The PAT control strategy was compared to a traditional manual control strategy using two different glucose feeding strategies—Low and High.

### 3.1 Model development for monitoring mammalian cell cultures

Five mammalian cell culture runs were cultured and harvested across a 14‐day period. The offline process parameters were recorded twice a day, once in the morning and once in the afternoon to ensure a larger sample range for model development. The offline measured performance for each of the runs is depicted in Figure 1. Bioreactor 3 did not achieve a high titre, due to the high accumulation of lactate and high base addition post‐inoculation, resulting in limited growth. It was still included in the dataset to increase model robustness to non‐optimal process conditions.

**FIGURE 1:**
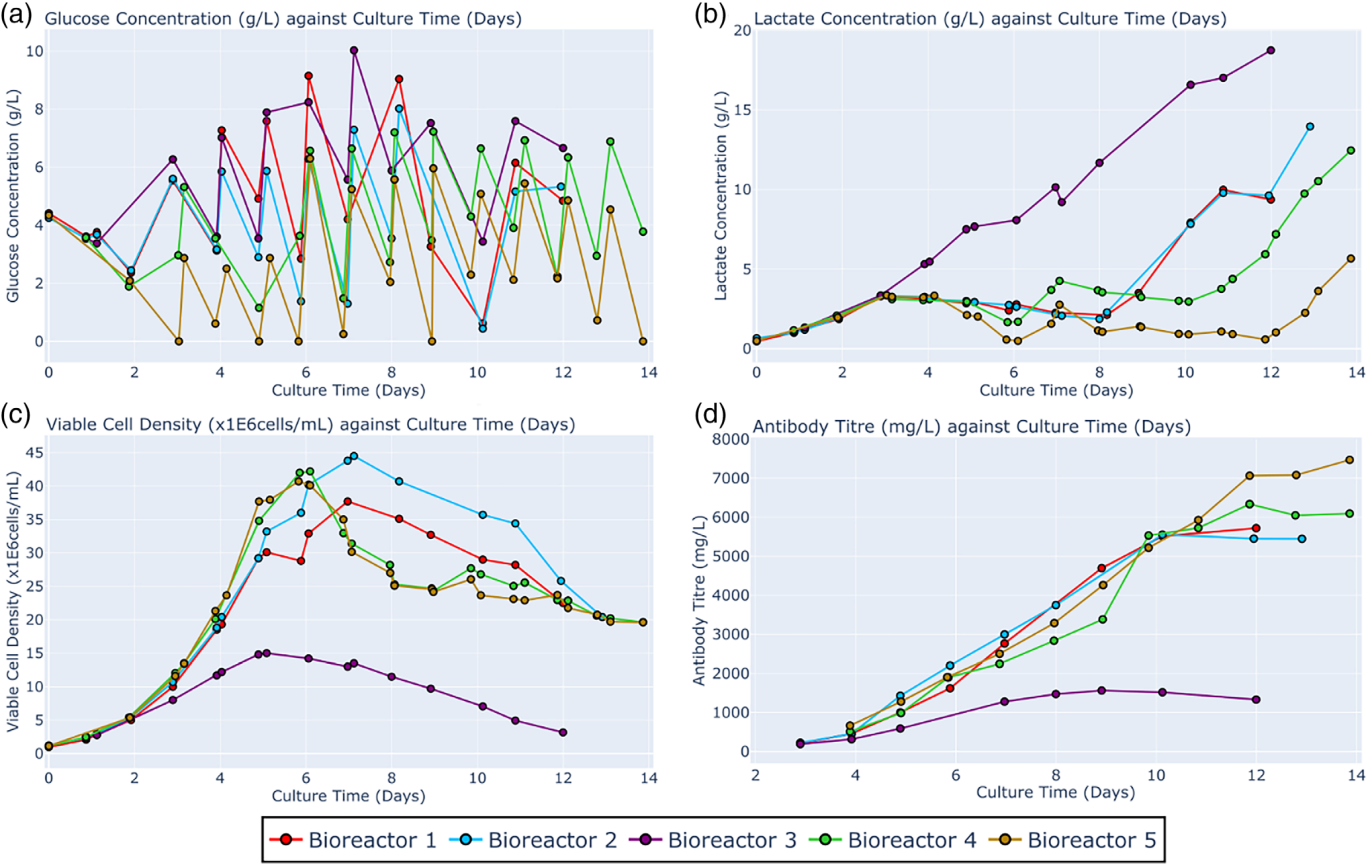
Time series graphs of offline analytical measurements from 7L mammalian cell cultures for (a) glucose concentration, (b) lactate concentration, (c) viable cell density (VCD), and (d) antibody titre.

Inline Raman spectra acquisitions were collected during sequential 12.5‐min intervals throughout the cell culture process. The raw spectra from the Bioreactor 2 run are depicted in Figure 2a, as a representative example. As the cell culture progressed, the baseline of the raw spectra increased. This increase was caused by the fluorescence background in the bioreactor. The regular increases in fluorescence corresponded to the timing of the two‐part nutrient‐dense feed additions to the culture. These feeds contained components which cells utilized for their metabolism and protein expression including large quantities of amino acids (^35^). One potential source of fluorescence in these feeds is the aromatic amino acids: tryptophan, tyrosine, and phenylalanine. These have been documented in the literature to fluoresce independently and as a structural component in proteins.^36^, ^37^ Fluorescence poses a significant challenge for Raman spectroscopy and by extension model building. Fluorescence causes a masking effect on Raman signals, resulting in signals with low intensity being dominated and becoming undetectable in the spectra.^38^ This is challenging in fed‐batch upstream processes, as accumulating fluorescence results in time‐dependent spectral features. Building models from these spectra impacts the assignment of features and increases model error. To minimize the impact on model performance, baseline correction techniques were employed to approximate the fluorescence in the spectrum and adjust the baseline accordingly. Following baseline correction, scatter correction algorithms were applied to normalize the baseline of the spectrum to increase spectra to spectra and run to run comparability for model building. This is critical when the spectral baseline and culture environment change throughout the run. A smoothing filter was applied to remove random noise while retaining the important Raman spectral features. Random noise can negatively impact model building and prediction performance due to the cumulative impact of small oscillations being magnified by model coefficients. An example of fully preprocessed spectra for the lactate concentration PLSR model for Bioreactor 2 is shown in Figure 2b. The specific preprocessing techniques and associated parameters utilized for each model are outlined in Table 1.

**FIGURE 2:**
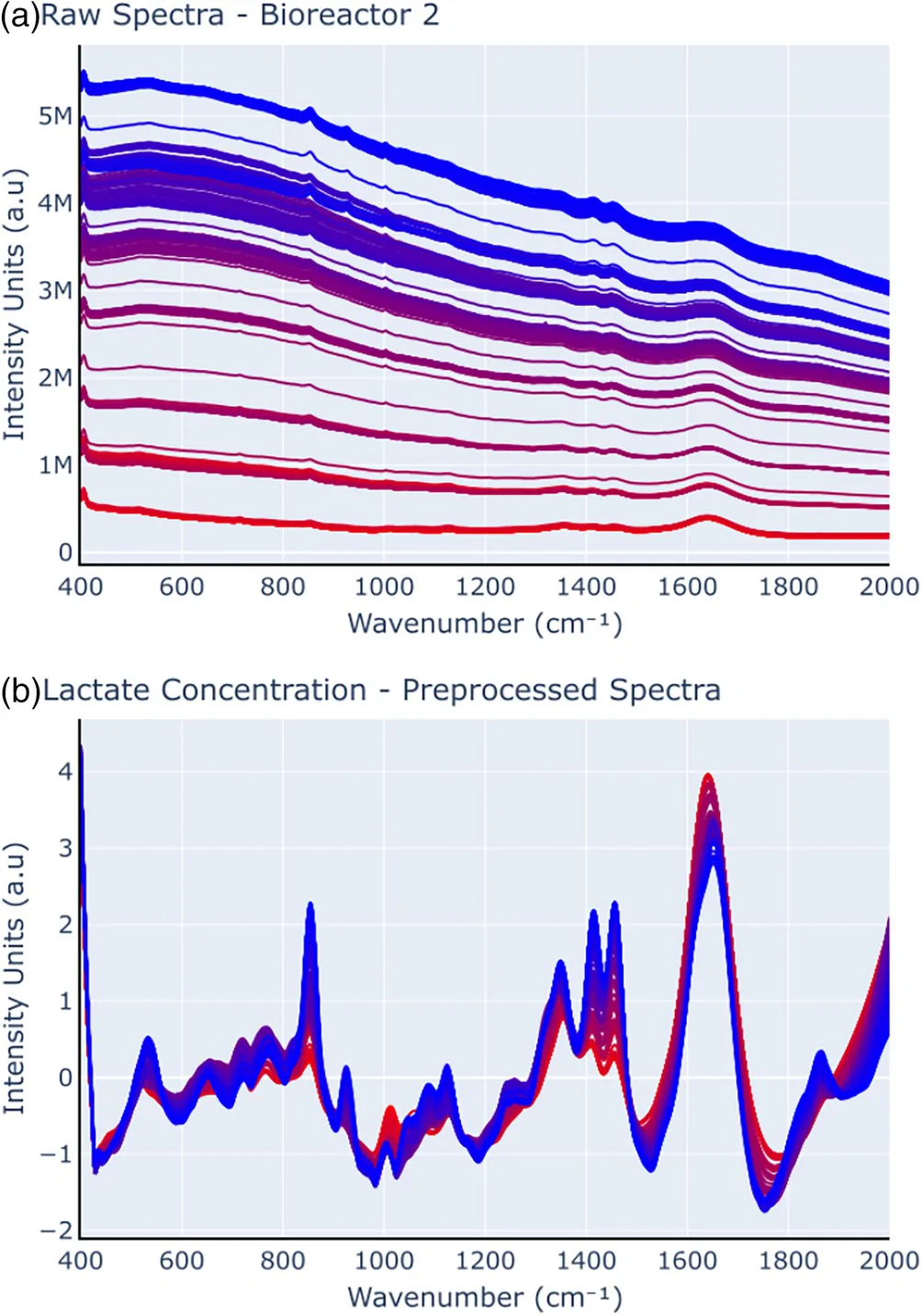
Inline Raman spectra recorded from a 7L mammalian cell culture system from Bioreactor 2 with (a) raw spectra and the (b) fully preprocessed spectra for the lactate concentration model. Spectra was recorded from day 0, inoculation day (red) to day 14, harvest day (blue).

The preprocessed spectra were used to develop and train several PLSR models to predict glucose concentration, lactate concentration, VCD, and antibody titre. The models were trained and optimized using Bioreactors 1, 3, 4 and 5 to identify optimal preprocessing and PLSR hyperparameters by reducing the average RMSE of a four‐fold cross‐validation method. Bioreactor 2 was set aside as a test set to assess the generalizability of each model on unseen data and model robustness. The model performance for each process parameter model is summarized in Table 2.

**TABLE 2:** Summary of the partial least squares regression (PLSR) model performance and statistics for glucose concentration, lactate concentration, viable cell density (VCD), and antibody titre.

Response variable	Dataset	Dataset Size	Coefficient of determination (*R* ^*2*^)	Root mean square error (RMSE)	Normalized root mean square error (NRMSE)
Glucose concentration (g/L)	Training	85	0.976	0.377	0.032
	Testing	18	0.955	0.487	0.066
Lactate concentration (g/L)	Training	84	0.990	0.322	0.010
	Testing	19	0.976	0.605	0.038
Viable cell density (×1E6 Cells/mL)	Training	86	0.957	2.305	0.065
	Testing	18	0.927	3.954	0.088
Antibody titre (mg/L)	Training	39	0.990	221.096	0.010
	Testing	9	0.968	362.088	0.042

A graphical representation of each PLSR model and its predictive accuracy compared to corresponding offline measurements is provided in Figure 3. The PLSR glucose concentration model, depicted in Figure 3a, demonstrated the capability to predict glucose concentrations with high accuracy up to 10 g/L. The RMSE for the training and testing sets was calculated as 0.377 and 0.487 g/L, respectively, corresponding to 3.73% and 4.82% of the total glucose concentration range investigated. These results are consistent with comparable applications of inline Raman spectroscopy process models reported in the literature. Webster et al. utilized inline Raman spectroscopy and PLSR to predict and control glucose concentrations in a fed‐batch mammalian cell culture, achieving a testing RMSE of 0.42 g/L.^39^

**FIGURE 3:**
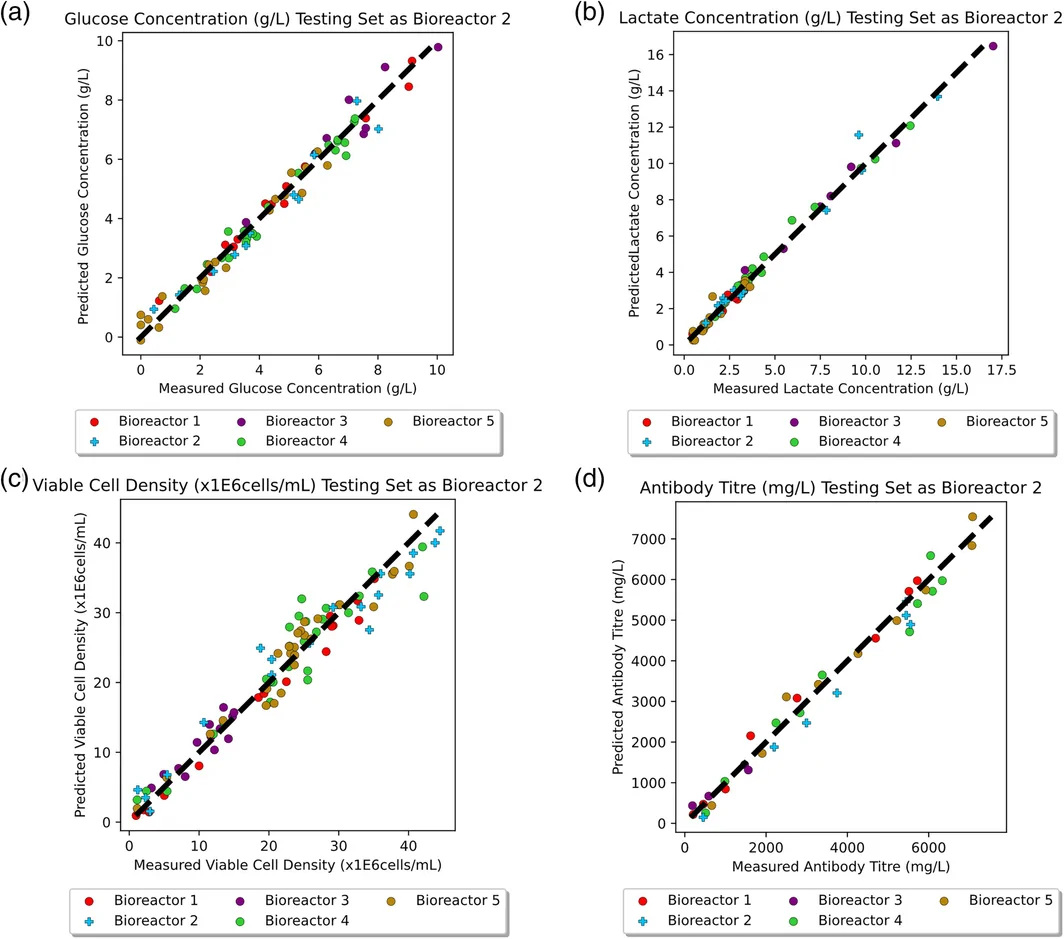
Parity graphs depicting the predictive performance of four partial least square regression (PLSR) models predicting (a) glucose concentration, (b) lactate concentration, (c) viable cell density (VCD), and (d) antibody titre. The PLSR models were trained and optimized using Bioreactors 1, 3, 4, 5 and tested on Bioreactor 2 to assess the generalizability performance.

The lactate concentration model presented in Figure 3b demonstrates the ability to accurately predict lactate concentrations up to 18 g/L. From Table 2, the model's performance on the training set yielded a RMSE of 0.322 g/L, while the testing set achieved an RMSE of 0.605 g/L, corresponding to total range percentage of 1.78% and 3.36%, respectively. In comparison, Matthews et al. utilized Raman spectroscopy and PLSR to predict lactate concentrations within a range of 0–5 g/L, resulting in a testing RMSE of 0.20 g/L across datasets from four different fed‐batch mammalian cell cultures.^23^ While the PLSR model described in this paper exhibits higher error in the testing set than the model by Matthews et al., this could be attributable to the differences in training ranges of lactate concentration. The PLSR model in this paper predicts lactate concentrations over a range nearly four times greater than Matthews et al., and as such, an increase in error is not unexpected. Both glucose and lactate models in this study produced errors which are comparable to the error of 0.5 g/L from industry‐standard offline analyzers. This suggests that these models could function as a replacement for these offline techniques with the added benefit of real‐time measurements.

The VCD PLSR model has been shown to accurately predict cell densities up to 45 × 1E6 cells/mL. As shown in Table 2, the associated model error was 2.305 × 1E6 cells/mL and 3.954 × 1E6 cells/mL for the training and testing set, respectively. These errors account for 5.30% and 9.08% of the total VCD ranges analyzed. As shown in Figure 3c, the model predicts VCD with greater accuracy at lower concentrations, implying that residual variance is not constant across the VCD range. A key factor contributing to this pattern is the sample preparation process, which required diluting all samples to below 10 × 1E6 cells/mL for compatibility with the cell counter. Since the dilution factor proportionally scaled the measured values, it also amplifies measurement error, leading to greater variability in the final reported values.

The antibody titre PLSR model accurately predicted the concentration of therapeutic protein up to 7.5 g/L (Figure 3d). With a training and testing error of 221.10 mg/L (3.03% of total range) and 362.09 mg/L (4.97% of total range) respectively. These errors were again comparable to models found in literature within both offline^40^ and inline^41^ Raman cell culture applications. The ability to monitor these key parameters in real‐time enables the development of more advanced control strategies for bench‐scale bioreactor systems. In addition to reducing time and labor costs from reduced sampling rates.

### 3.2 Feedback control strategy workflows

To evaluate the impact of real‐time glucose monitoring on process control, two distinct feedback control strategies were implemented: a manual control strategy and the PAT‐based control strategy. The manual control method relied on infrequent offline sampling and reactive bolus glucose additions, which led to fluctuations in glucose concentration and suboptimal culture conditions. In contrast, the PAT control strategy utilized inline Raman spectroscopy to predict glucose concentrations in real‐time, enabling automated and adaptive glucose feeding. Figure 4 outlines the workflows of both control strategies, highlighting their key differences in sampling frequency, data integration, and glucose addition mechanisms. Glucose targets were comparable in both control modes.

**FIGURE 4:**
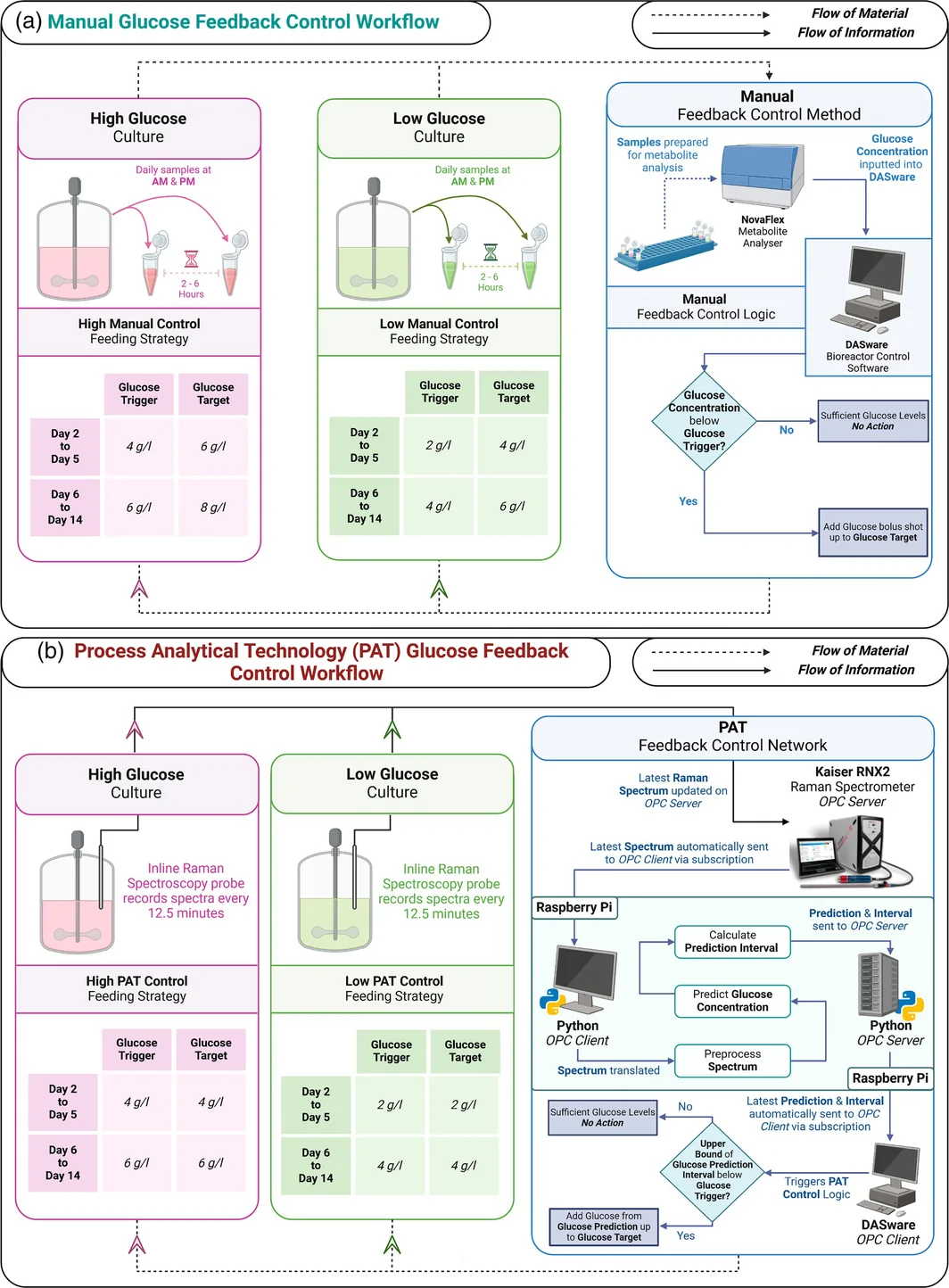
Workflow of the feedback control strategies for (a) Manual control and (b) process analytical technology (PAT) control.

Figure 4a illustrates the manual control strategy workflow, a commonly employed control strategy for glucose in mammalian cell cultures. Each bioreactor was sampled twice a day, with intervals of 2–6 h between samples. These samples were manually analyzed using an offline metabolite analyzer and the corresponding offline glucose measurement was manually inputted into the bioreactor control system. If the offline glucose reading was below the glucose trigger point, a glucose bolus shot was administered to reach the glucose target. This control strategy is limited by the number of samples the operator can complete, resulting in suboptimal monitoring. Fewer sampling points can result in a slower response time to glucose demand, increasing the risk of glucose starvation and batch failure. To compensate for infrequent sampling, the glucose target was set at 2 g/L higher than the glucose trigger to reduce this risk between sample points.

Figure 4b illustrates the PAT control strategy workflow, which enabled the near‐instant data exchange between bioreactor and PAT sensor via OPC‐UA. The PAT sensor (Raman spectrometer), bioreactor control system, and Raspberry Pi were connected across a high‐security internal lab network. The Raman system continuously updates its OPC‐UA server with the latest Raman spectrum, which is automatically queried and retrieved by the Raspberry Pi OPC‐UA client in real‐time through a subscription mechanism. The Raspberry Pi, running both an OPC‐UA client and server, processes incoming spectral data within milliseconds, preprocessing and translating it into a predicted glucose concentration along with 99% prediction interval bounds using the stored glucose model. This processed information was immediately made available to other network devices via the Raspberry Pi OPC‐UA server. The bioreactor control system, through its OPC‐UA client, automatically read the updated glucose prediction and interval bounds and stores the data internally. Upon retrieval, the system triggered the PAT control logic, ensuring real‐time glucose control within the bioreactor. A prediction interval‐based approach defined in Section 2.8 was employed to incorporate the uncertainty of the model into the prediction estimates. If the upper bound of the prediction interval fell below the glucose trigger point, glucose was added from the predicted concentration to the target. The upper bound was selected to minimize the risk of glucose overfeeding and to account for increased prediction oscillations over time caused from fluorescence masking. Due to the increased frequency of measurements every 12.5 min better control was enabled. The glucose target was the same as the glucose trigger for both feeding strategies.

These control frameworks were successfully implemented within a laboratory‐scale environment. However, additional challenges must be addressed when deploying PAT‐based frameworks within a GMP manufacturing setting. Robust mitigation strategies and fallback procedures for offline sampling are required to ensure process continuity in the event of communication failures between PAT and control systems. Furthermore, all data handling, storage, and system integration must comply with 21 CFR Part 11 requirements to ensure data integrity and regulatory compliance within GMP operations.

### 3.3 PAT and manual glucose feedback control strategies

In this section, the glucose model built in Section 3.1 was deployed into a mammalian cell culture manufacturing system using the PAT control framework defined in Section 3.2. This PAT control strategy was compared to a traditional manual control strategy to assess its impact on culture performance including other process parameters and glycation. The comparison was assessed through using the control strategies with two different glucose feeding strategies: the Low feeding strategy and High feeding strategy.

The predicted glucose concentrations and the corresponding offline glucose concentrations for each PAT control run are illustrated in Figure 5. Throughout the process, offline glucose concentrations were maintained within the 99% prediction interval of the glucose model. Between day 10 and day 12, the system experienced a hardware issue with the Raspberry Pi overheating which resulted in a period of disconnect in the High and Low PAT control strategies as depicted in the blue‐shaded region in Figure 5a,b. This was subsequently resolved with the addition of heat sinks and cooling fans to improve temperature control.

**FIGURE 5:**
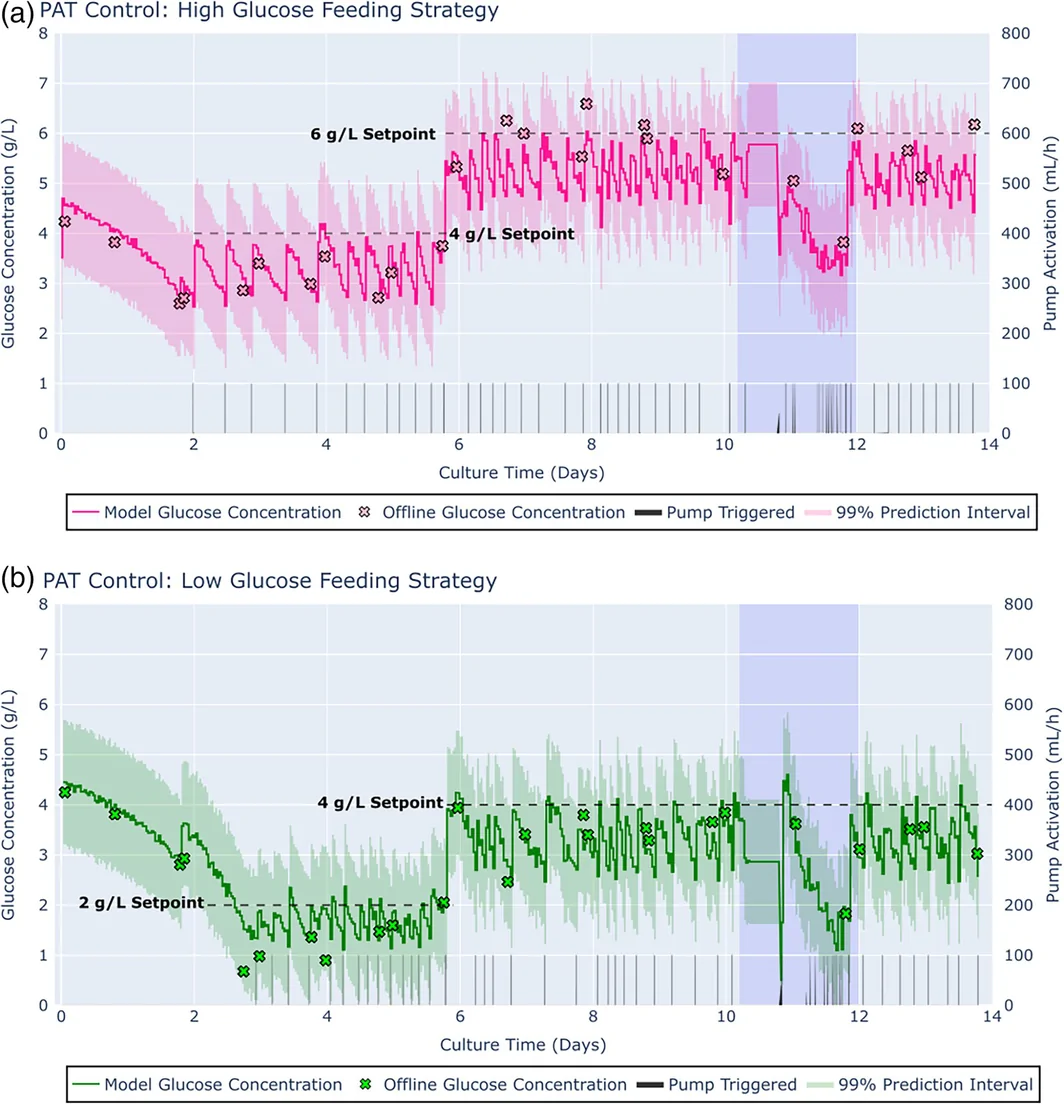
Glucose concentration times series plots depicting the process analytical technology (PAT) feedback control strategy at the (a) High glucose feeding strategy (Pink) and (b) Low glucose feeding strategy (Green). The black dotted lines depict the glucose trigger point for each glucose feeding strategy. The blue‐shaded region represents a period where the control loop was disconnected from the process due to hardware issues.

Figure 5a shows the culture model predictions and offline measurements for the 14‐day High PAT control strategy. When the control strategy was active, the glucose concentration was maintained on average at 3.15 g/L ± 0.39 g/L between day 2 and day 5 and 5.83 g/L ± 0.46 g/L between day 6 and day 14. On all days, the glucose concentration was consistently maintained slightly below the targets of 4 and 6 g/L for the High PAT control strategy, with very small deviations. This slight offset from the setpoint was intentional, as the controller was conservatively configured to minimize the risk of glucose accumulation and overfeeding while maintaining stable glucose control through the 99% prediction interval. This demonstrated that the control strategy can accurately control glucose at a high concentration of glucose.

The Low PAT control strategy for the 14‐day cell culture process is illustrated in Figure 5b. The offline glucose concentration samples slightly deviated from the predicted glucose concentration on day 2 to day 4, however, these deviations were accommodated in the control strategy as they were encompassed within the prediction intervals range. While the PAT control strategy was active, the glucose concentration was maintained on average at 1.29 g/L ± 0.47 g/L between day 2 and day 5 and 3.43 g/L ± 0.39 g/L between day 6 and day 14. The control strategies consistently maintain the glucose concentration just below the 2 and 4 g/L targets, respectively, with small deviations. Indicating that the control strategy can supplement and accommodate for deviations in model predictions while maintaining the desired glucose target.

The pump for the addition of glucose was activated 38 and 39 times in total over the 14 days for the High and Low PAT control strategy, respectively. This was an almost three‐fold increase compared to the manual control method, which was triggered 13 times across both feeding strategies. Furthermore, the frequency of pump activations increased as the run progressed, considerably after day 6, indicating that the demand for glucose varied throughout the culture. From day 6 onward, the period aligned with the expected peak VCD and the main phase of protein expression. This indicated that the increased glucose demand may be linked to the number of viable cells in the culture. This was expected as glucose plays a significant role in the metabolism of CHO cells, such as in oxidative phosphorylation, where the production of adenosine triphosphate is critical for both cell growth and protein expression.^42^

The comparison of the offline glucose concentrations for both the PAT control and manual control strategies with the High and Low glucose feeding strategy is depicted in Figure 6a,b, respectively, alongside the performance metrics in Table 3. The average offline glucose concentration of the High Manual control in Figure 6a was 3.56 g/L ± 1.51 g/L from day 2 to day 5 and 4.96 g/L ± 2.03 g/L between day 6 and Day 14. The average offline glucose concentration of the Low Manual control depicted in Figure 6b was 1.48 g/L ± 1.42 g/L from day 2 to day 5 and 3.67 g/L ± 2.24 g/L between day 6 and day 14. While the average offline glucose concentrations are similar across control strategies, the greatest difference lies in the standard deviation and variance of these concentrations. There are larger oscillations in offline glucose concentration in the manual control workflow in comparison to the PAT control method. These drastic changes in the culture glucose environment have been reported to impact metabolic regulation and glucose uptake.^43^

**FIGURE 6:**
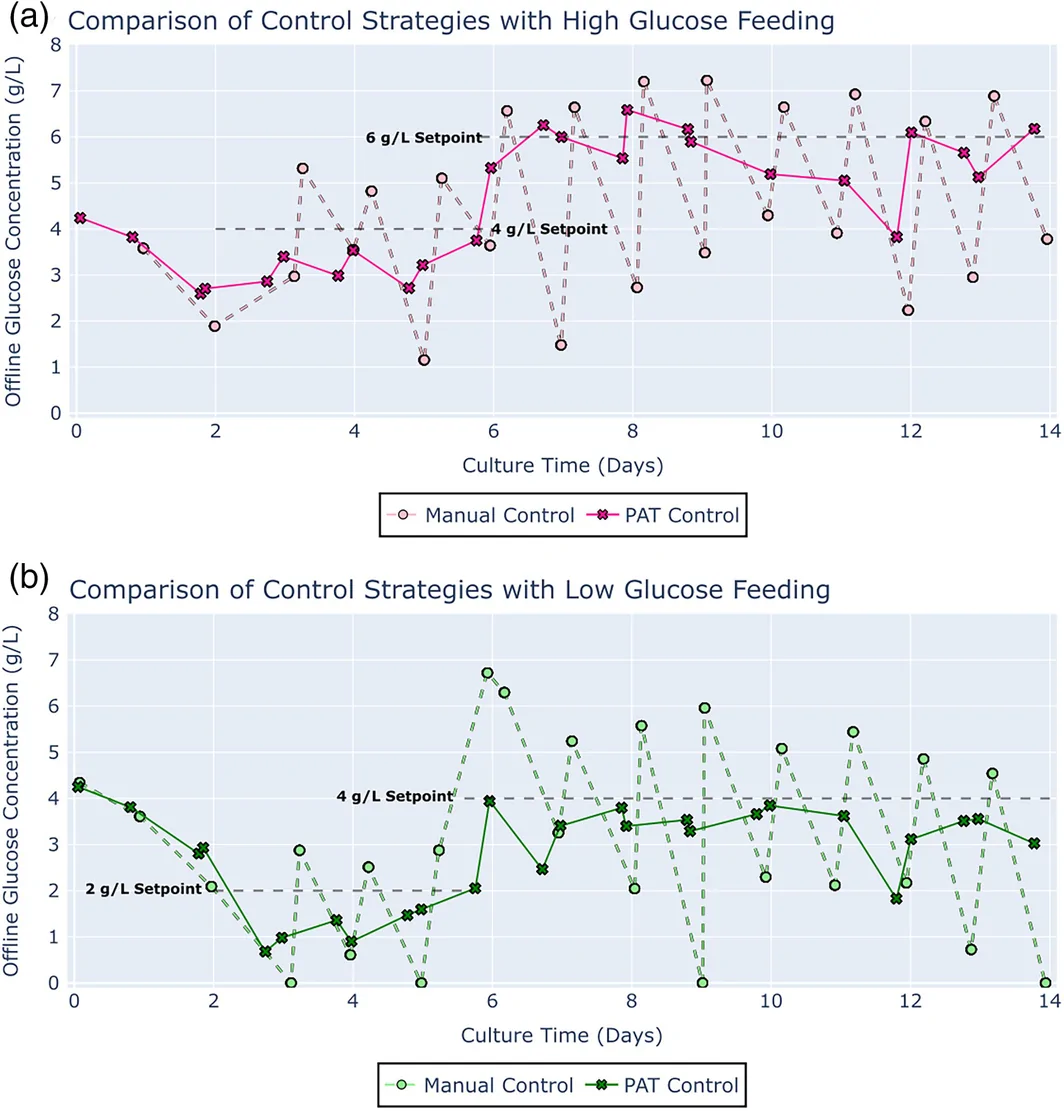
Time series plots of offline glucose concentration comparing different control strategies for (a) High glucose feeding strategy (Pink) and (b) Low glucose feeding strategy (Green). The black dotted lines depict the glucose trigger point for each glucose feeding strategy. The manual feedback control strategy (Manual Control) is depicted with circular symbols, and the process analytical technology (PAT) feedback control (PAT control) strategy is depicted with cross symbols.

**TABLE 3:** Summary of performance metrics for the different feeding and control strategies.

Control strategy	Coefficient of determination (*R* ^2^)	Root mean square error (RMSE) (g/L)	Days 2–5 average glucose concentration (g/L)	Days 6–14 average glucose concentration (g/L)	Glucose concentration range (g/L)
High PAT control	0.967	0.243	3.15 ± 0.39	5.83 ± 0.46	2.60–6.59
High manual control	‐	‐	3.56 ± 1.51	4.96 ± 2.03	1.16–7.23
Low PAT control	0.959	0.214	1.29 ± 0.47	3.43 ± 0.39	0.68–4.25
Low manual control	‐	‐	1.48 ± 1.42	3.67 ± 2.24	0.00–6.30

The manual control method, while averaging close to the ideal setpoint, struggled to consistently maintain the glucose setpoint due to limited sampling frequency. This inconsistency stems from its reliance on predefined parameters, which do not account for additional process‐related information. As shown in Figure 6b, the Low manual control method frequently failed to maintain the glucose setpoints, resulting in multiple periods of glucose depletion within the bioreactor, particularly on days 3, 5, 9, and 14. Extended periods of glucose starvation are suboptimal, as they can lead to cell apoptosis.^44^, ^45^ Furthermore, glucose demand fluctuated throughout the process, and it was evident that the manual control strategy was unable to adapt to these variations in a timely or effective manner. Incorporating other process‐related information into the control strategies would reduce the impact of glucose demand while preserving the limited sampling frequency. Rowland‐Jones et al. proposed a glucose control strategy by using an offline Raman spectroscopy microscope to predict VCD and glucose concentration, from as low a volume as 25 μL samples taken from a miniature fed‐batch bioreactor system.^46^ They proposed adapting the feeding rate of both the glucose concentration and the nutrient‐dense feed based on the predicted VCD to facilitate continuous feeding of glucose.

To assess the variance between control strategies, the Fligner–Killeen test for homogeneity of variances was applied, and the Mann–Whitney U test was used to compare the means of the control strategies.^47^, ^48^ Each control strategy dataset was randomized before analysis. These tests were selected due to the non‐normal distribution of the offline glucose concentrations. The analysis indicated insufficient evidence to suggest a significant difference (*p* >0.05) in the mean offline glucose concentrations between the manual and PAT control methods across all feeding strategies. However, a significant difference (*p* <0.05) in the variance of glucose concentrations was observed between the control strategies from day 6 to day 14, indicating that the PAT control method reduced variability. These findings suggest that glucose concentrations were more consistently maintained with the PAT control method compared to the Manual control strategy in this period. Further, while the Manual control strategy was sufficient to maintain glucose concentrations during the early stages of cell culture, it was less effective in managing the increased demand during the later stages. Overall, this highlighted that variance, in conjunction with mean, is a more representative metric of an effective process control.

### 3.4 Comparative analysis of a representative upstream process under PAT and Manual glucose control approaches

A comparison of other CPPs and CQAs between the different control and feeding strategies is illustrated in Figure 7. Figure 7a shows that the PAT control strategy was able to achieve higher VCDs, with a peak of 53 × 1E6 cells/mL in comparison to the manual control strategy which could reach a maximum of 42 × 1E6 cells/mL. Furthermore, the PAT control method maintained these higher VCDs for longer compared to the manual control which started to decline from day 6 onwards. The Low PAT control strategy achieved the highest VCDs across the runs, suggesting that consistently maintained lower glucose levels encourage a higher maximum VCD and increased longevity in the process. Additionally, this suggests that the PAT controlled process could be even extended beyond day 14 to achieve higher biotherapeutic product yields. The cell viability throughout the runs maintained a similar trend between feeding strategies as shown in Figure 7b. The largest difference occurs between the control strategies with the PAT control maintaining cell viability above 80% compared to manual control which was below 60% by the day of harvest. This suggests that the glucose feeding method had more of an impact on cell death than the actual glucose concentration.

**FIGURE 7:**
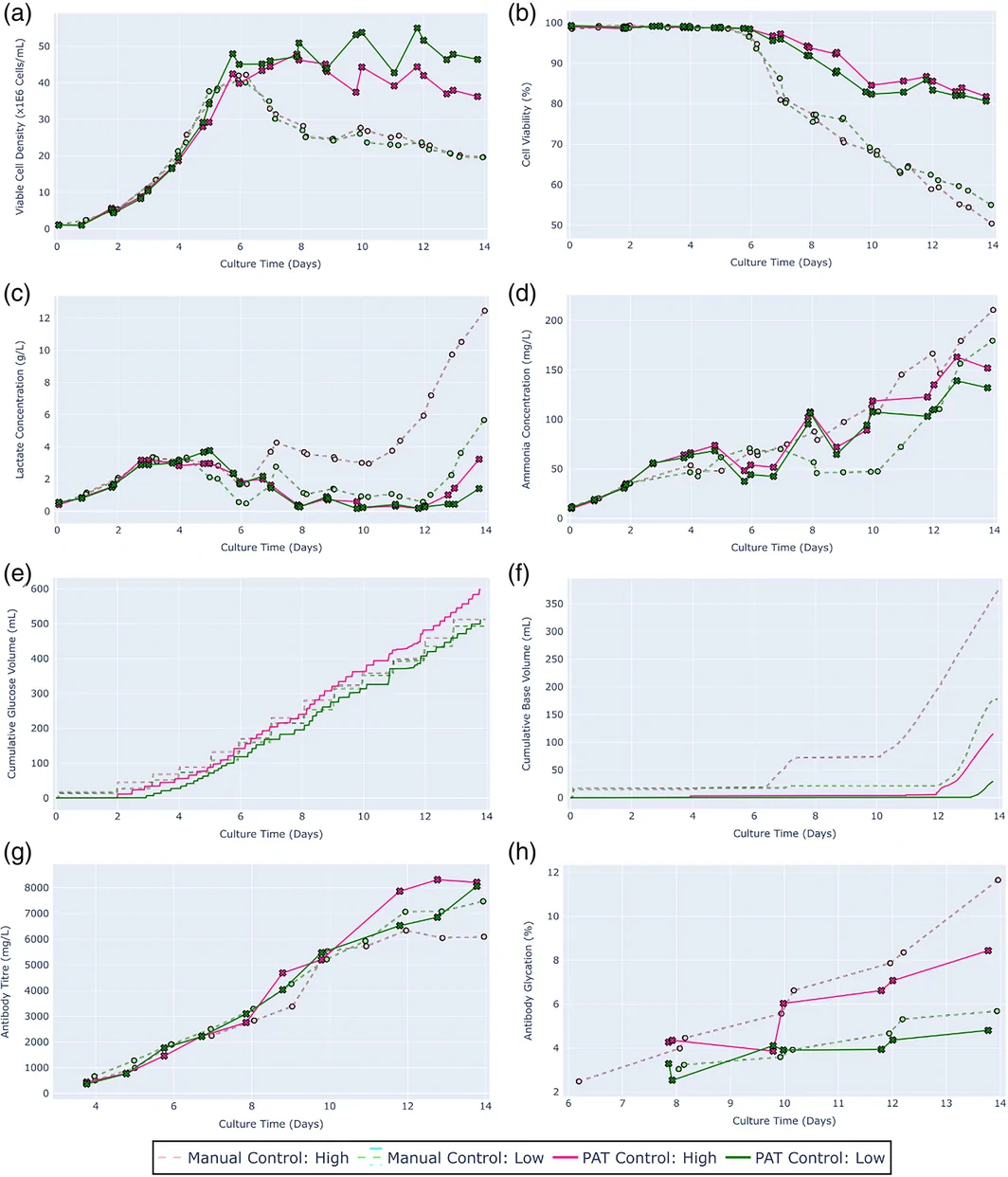
Comparison of time series bioreactor parameters and measurements across different glucose control strategies for (a) viable cell density (VCD), (b) cell viability, (c) lactate concentration, (d) ammonia concentration, (e) cumulative glucose feed addition, (f) cumulative base addition, (g) antibody titre, (h) antibody glycation percentage. The manual feedback control (Manual Control) strategy is depicted in a dashed line and the process analytical technology (PAT) feedback control (PAT Control) strategy is depicted with a solid line. The High glucose feeding strategy is depicted in a pink and the Low glucose feeding strategy is depicted in green.

There was a decrease in lactate accumulation between the PAT control and the manual control strategies, as seen in Figure 7c. This suggests that the manual control strategy promotes a higher secretion of lactate compared to the PAT control strategy. It was observed that the feeding strategy impacts lactate accumulation with a higher glucose setpoint resulting in increased lactate accumulation. This has been documented in literature, where higher levels of glucose concentration in fed‐batch bioreactors affect lactate production.^49^ The PAT control produced lower end point amounts of ammonia in comparison to the manual control in Figure 7d. It was observed that the manual control experienced a similar trend to the PAT control until day 10, where the ammonia production rapidly accelerated from 47.2 and 112.89 mg/L up to 179.58 and 210.64 mg/L at harvest for the Low and High feeding strategies, respectively.

As illustrated in Figure 7e, the total glucose volume added across all control and feeding strategies was comparable. Further, it was observed that the amount of glucose added through the manual control strategy between bolus additions was comparable to the respective PAT control strategy for the same period. This deviated only for the High PAT control strategy, where the amount of glucose added exceeds the High manual control. This increased uptake in glucose could be due to the increased expression of product within the same period between day 10 and day 12, as seen in Figure 7g.

Figure 7f illustrates that the overall base addition is decreased across both feeding strategies for PAT control compared to the manual control. This correlated well with the lower lactate levels in the PAT controlled processes. As shown in Figure 7g, the PAT control strategy resulted in an up to 35% observed increase in antibody titre across all feeding strategies in comparison to the manual control method. The increased yields could be attributed to the increased VCDs and cell longevity. It clearly demonstrates the potential of PAT control for optimizing processes and could be deployed where a product is more difficult to express.

As shown in Figure 7h, the quality profile of the expressed biotherapeutic product demonstrated up to a 27% observed reduction in glycation across all runs when comparing the PAT control strategy to the Manual control strategy. This suggests that glucose concentration levels and the control strategy both impact therapeutic protein glycation levels, with drastic changes in glucose concentration at a high concentration setpoint increasing variability within the product's structure.^50^ This can be attributed to the increased availability of glucose reacting with the excreted product within the media. While higher glucose levels can be attributed to higher antibody titre, the variability post‐translational modification increases, resulting in a more heterogeneous product. Additionally, high levels of glycation in therapeutic proteins can impact stability^51^ and potentially lead to the development of advanced glycated end products,^52^ impacting the efficacy and pharmacokinetics as a treatment option.^53^ It is important for industry to consider the amount of protein expressed against the consistency in product characteristics.

The cell line chosen for these runs exhibited undesirable process characteristics under the manual control strategy, such as end‐point lactate accumulation, low cell viability, and high levels of glycation in the product. However, these findings suggest that the control and feeding strategy have a significant impact on the cell and overall cultures' performance. With the Low PAT control strategy, the cell line had reduced undesirable characteristics that transformed a poor‐performing cell clone into a viable and competitive candidate. Furthermore, this information could have applications at earlier bioprocess stages, such as in cell line development where poor‐performing clones could be made viable by adapting the feeding and control strategy.

## 4 CONCLUSION

This study demonstrated the successful application of inline Raman spectroscopy for real‐time monitoring and/or control of process parameters in a mammalian cell culture system for biotherapeutic production. By leveraging advanced preprocessing techniques and PLSR, Raman spectroscopy was capable of predicting glucose concentration, lactate concentration, VCD, and antibody titre with high accuracy. The developed glucose prediction model was integrated into a robust and standardized PAT control strategy. This approach facilitated the communication and exchange of information between 3 systems through an OPC‐UA framework: a PAT sensor (Raman Spectrometer), a Raspberry Pi and bioreactor control system. The PAT control was evaluated against a manual control approach under two distinct glucose feeding strategies. The results showed that the PAT control strategy improved glucose regulation, reducing variability and maintaining setpoints more consistently compared to the manual control method. This led to improvements in cell culture performance, including increased VCD, prolonged cell viability, and reduced lactate and ammonia accumulation. Additionally, the PAT control method observed an up to 35% increase in product titre and up to 27% reduction in glycation, highlighting its potential for improving both product yield and quality. Notably, the Low PAT control strategy exhibited the optimal culture performance, suggesting that tightly controlled low glucose microenvironments can improve metabolic efficiency and process longevity. While these results demonstrate the potential of Raman‐enabled PAT glucose control, additional bioreactor runs are required to evaluate the generalizability and reproducibility of these findings across broader process conditions.

Overall, this study demonstrates the value of Raman spectroscopy as a powerful tool for bioprocess monitoring and control. The findings suggest that integrating real‐time predictive modeling with automated control strategies can significantly enhance process stability, reduce operational risks, and improve product quality consistency. For the standardized framework to be GMP compliant, additional considerations for data integrity and cybersecurity need to be explored in future. This may require close collaboration with commercial vendors that meet the necessary compliance and regulatory standards. Once these integration challenges are addressed, the opportunities for real‐time control will enable much tighter regulation of CPPs and CQAs, allowing for higher productivities and streamlined downstream processing operations that ultimately accelerate process development timelines and transform the sector.

## AUTHOR CONTRIBUTIONS

**Noah Hitchcock:** Resources. **Rajesh Mistry:** Resources. **Richard Turner:** Funding acquisition. **Daniel Ray:** Methodology; investigation. **Fabio Zurlo:** Methodology; investigation. **Matthew Cheeks:** Writing – review and editing; supervision; project administration. **Jonathan Welsh:** Investigation; methodology; writing – review and editing; supervision; conceptualization; resources; project administration. **Kasia Kozakowska‐McDonnell:** Methodology. **Irfan Ahanger:** Methodology; investigation. **Matthew Banner:** Methodology; visualization; writing – original draft; writing – review and editing; software; formal analysis; conceptualization; investigation; validation; data curation. **Suzanne S. Farid:** Supervision; funding acquisition. **Michael Thomas:** Writing – review and editing; supervision; validation; funding acquisition; project administration. **Stephen Goldrick:** Conceptualization; funding acquisition; writing – original draft; writing – review and editing; methodology; validation; supervision; formal analysis; project administration.

## CONFLICT OF INTEREST STATEMENT

The authors declare no conflicts of interest.

## Data Availability

The data that support the findings of this study are available from AstraZeneca. Restrictions apply to the availability of these data, which were used under license for this study. Data are available from the author(s) with the permission of AstraZeneca.
